# Challenges of Eye Health Care in Children and Strategies to Improve Treatment Uptake: A Qualitative Study from the Perspective of Eye Care Professionals in the UK

**DOI:** 10.22599/bioj.133

**Published:** 2019-05-14

**Authors:** Viola Cassetti, Tom Sanders, Alison Bruce

**Affiliations:** 1 University of Sheffield, GB; 2 Northumbria University, GB; 3 Bradford Institute for Health Research, GB

**Keywords:** Child Health, Health Professionals, Screening, eye health care, treatment uptake

## Abstract

Follow up from universal vision screening at four to five years has been shown to be low in England, potentially increasing the risk of vision disorders not being treated. This study explores vision specialists’ views on the perceived barriers and facilitators encountered when engaging with parents and young children, and the strategies adopted to improve child/parent centred care. Fifteen semi-structured qualitative interviews were conducted with eye care professionals to explore perspectives on the challenges of treating children. Thematic analysis was performed to identify key barriers and the strategies eye care professionals adopt to enhance person-centred eye care when working with young children and their families. Two overarching themes were identified related to the professional-patient relationship. The first reflects the challenges which vision specialists experience when treating children, considering lack of eye health education and negative attitudes to diagnosis and treatment as major barriers. The second discusses the strategies adopted to tackle those barriers. Three strategies are proposed to enhance child-centred eye care: more eye health education, more personalised communication to enhance referral uptake and the development of better coordinated pathways of care between schools, communities and hospital services.

## Introduction

Screening children’s vision at a young age is recommended to detect signs of potential disorders such as amblyopia, strabismus and refractive error, which can affect the child’s visual development. Early treatment of reduced vision is particularly important in children as treatment is age-sensitive and should be started as early as possible (de Zoete 2007). In the UK, the National Screening Committee (2013) recommends screening children aged four to five years for vision disorders by an orthoptist-led service. In England, vision screening is undertaken in the first year of school. Children who fail the assessment are referred to Hospitals or community services for further tests (Public Health England 2017). Research has shown that attendance at follow-up appointments is relatively low. A recent study conducted in England (Bruce & Outhwaite 2013) found approximately 70% of children attended follow-up after school screening and the British and Irish Orthoptic Society (BIOS) 2016–17 audit of vision screening reports a mean attendance of 70% ranging widely from 27% to 95% (Griffiths et al. 2018).

Poor vision has been reported to impact on educational attainment (Bruce et al. 2016), thus exploring the main challenges in childhood eye care is important.

The existing literature on missed appointments in adult patients suggests there may be a variety of factors affecting uptake rates, such as lack of awareness of the importance of eye care, conflicting family needs, socio-cultural background or economic conditions (Shickle & Farragher 2014; Patel et al. 2006; Gower et al. 2013; Touch and Berg 2016).

Reviewers of the National Committee Screening recommendations (Solebo et al. 2013) have expressed concern over the lack of robust evidence on whether the current vision testing, screening and follow-up pathway in the UK is acceptable to young children and their families. A review of the published literature was carried out to explore current challenges in vision services for children and uptake of referrals and treatments. However, most of the literature reported findings from non-UK settings, and the few publications related to childhood vision care in the UK did not explore barriers to service uptake and treatment compliance (Alexander et al. 2009; Little & Saunders 2015; Bruce & Outhwaite 2013; Solebo et al. 2013; Solebo et al. 2015; Toufeeq & Oram 2014).

Currently, in England, when children fail vision screening at school, a letter is sent home inviting the parent or carer to attend a follow-up eye examination with their child either at a hospital (presence of strabismus or dense amblyopia) or a high-street optometrist (community service) of their choice (mild to moderate vision reduction), depending on the vision problem detected. Public Health England (2017) provides more detailed information regarding the diagnostic pathway.

In the area where this study was undertaken, when children are referred to community services, parents can choose which optometrists to see, and the optometrist will then send the results to the lead professional in vision screening services. However, if the child fails to attend the community optometrist, the lead professional for screening may be uncertain as to whether the child has attended or not.

**Figure 1:**
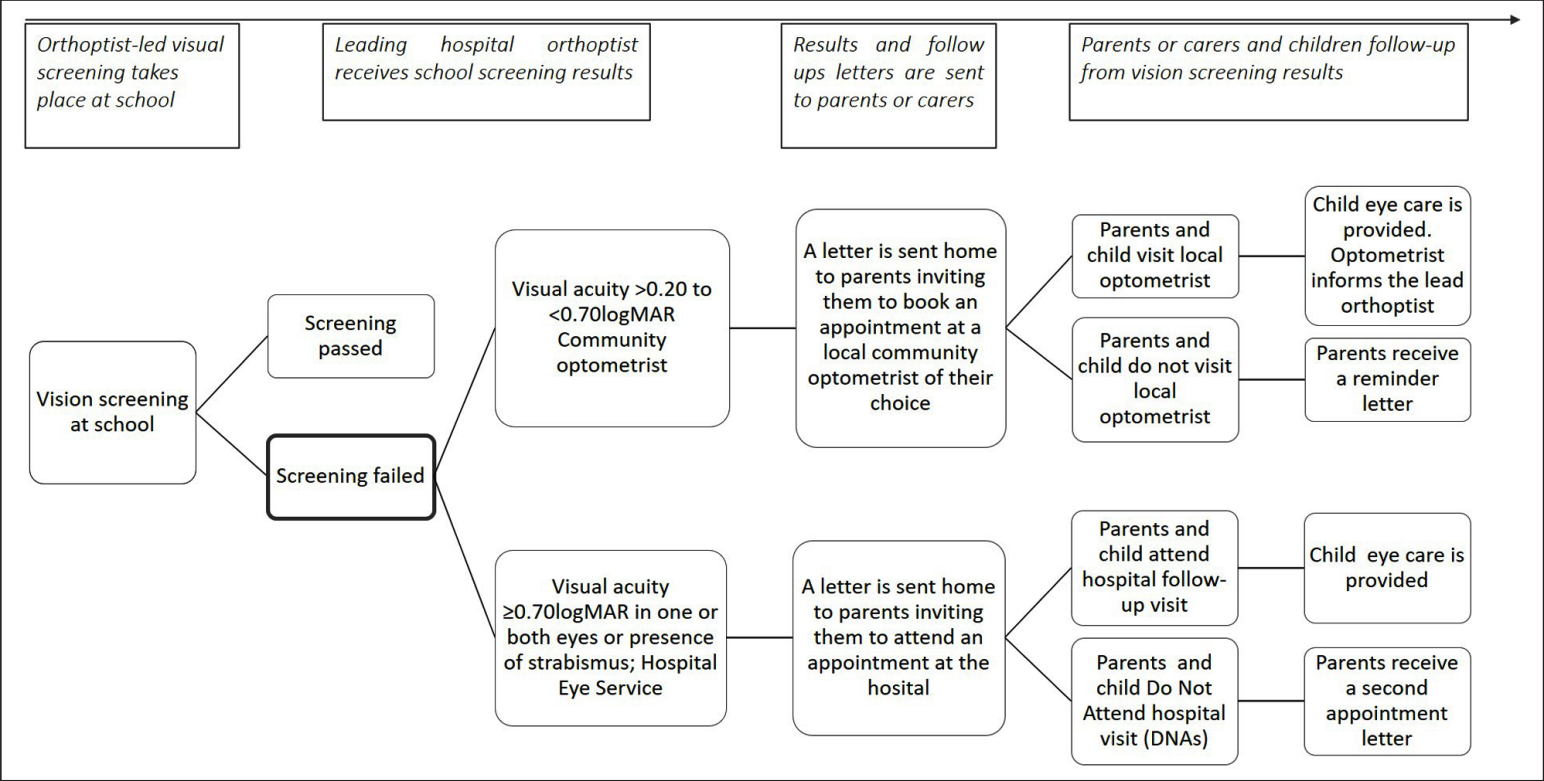
Local pathway of children post-screening vision service.

Figure 1 illustrates the main steps in the current local model of eye care.

The duration of the visit changes depending on the setting and this can influence families’ engagement in eye care. In hospitals, the eye examination can take up to two hours with the child being assessed by a number of eye professionals. The time-burden and the multiple procedures (Figure 1) required in the post-screening care may overload both children and carers, affecting their willingness to attend follow-up visits or to access future eye care services. On the other hand, community optometrists generally have 20-minute appointments with the child which the optometrists find challenging as certain tests can last more than thirty minutes, resulting in the need to re-book the child for a second appointment for which they cannot claim payment, as argued by some of the professionals in this study.

Given this scenario, and the low uptake of follow up for children vision care (Bruce & Outhwaite 2013), this study aimed to explore vision specialists’ views on the current model(s) of eye care and the perceived barriers and potential enablers to follow-up referrals and treatment when working with young children. It is part of a larger study on improving childhood vision care in Northern England, which included a qualitative study exploring carers’ perspectives on children’s vision services (Bruce et al. 2018). This research was approved by an Ethics Committee at the School of Health and Related Research, University of Sheffield.

## Methods

Fifteen qualitative semi-structured interviews were conducted between November 2015 and March 2016 with a convenience sample of vision specialists working in England: community optometrists (n = 4), hospital optometrists (n = 3), orthoptists (n = 5), ophthalmologists (n = 2) and one university professor. Written informed consent was obtained for each participant. The interviews included questions which aimed to explore professionals’ views on current childhood vision care services. In order to ensure that the views of every participant were captured within the semi-structured interviews, a topic guide was used covering challenges in current children’s vision care, and the strategies which could enhance a more patient-centred care approach when working with young children and their families. Interviews were audio-recorded and transcribed verbatim for analysis. Thematic analysis using N-Vivo 10 was conducted, initially coding line by line and then synthesising codes together in themes and sub-themes using constant comparative methodology (Strauss & Corbin 1998).

## Results

**Figure 2:**
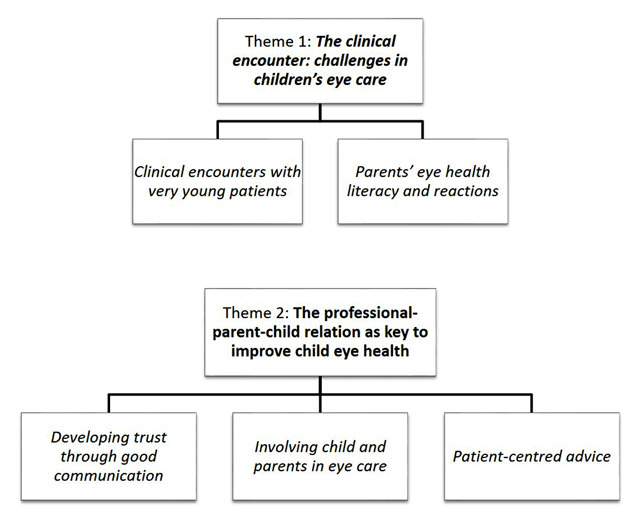
Themes and sub-themes identified through thematic analysis.

Two major themes were identified through the thematic analysis, as shown in Figure 2. The first theme related to the challenges encountered when treating children compared to adult’s eye care and the second to the strategies adopted to tackle those barriers.

The themes provide insights into how interactions of vision care specialists with children’s and carers’ lay beliefs may influence attitudes about eye health and treatment.

Additional information was synthesized to provide contextual information regarding the current model of children eye care.

### Challenges in Children’s Eye Care

#### Clinical encounters with very young patients

Treating young children is not the same as treating adults. Children can struggle during the visit itself, as

*‘a child can’t really tell you what they feel, what things look like. So, it does take a lot more work to get the information you need. And you also have the parents there, so you have an extra pair of eyes watching what you are doing’ [P11]*.

Children can also

*‘become bored straight away; adults just become angry but then they are very easy to examine when you see them. But children get tired or bored or hungry or upset or any of these things and then you can’t examine them easily’ [P5]*.

It can result in an incomplete assessment in one session because of their attention-span and this requires to re-book a second appointment with the child

*‘and they are a bit more familiar and might be in a better mood’ [P15]*.

Most professionals agree that seeing children may require multiple visit;

*‘There is a lot of chatting on that first visit. So, it will take two to three visits, not only to get all the information, but also for the child to be happy to see you, and to realise that it’s not going to be too dreadful’ [P11]*.

Some professionals also found the use of the eye drops problematic,

*‘drops sting quite a lot. You might make yourself unpopular by [using] them’ [P6]*,

and prefer to have someone else putting the drops in,

*‘which is usually a nurse or a healthcare assistant, simply because then they don’t see us as the “baddy”’ [P1]*.

#### Parents’ eye health literacy and reactions

Vision specialists claimed that parents’ poor attendance for follow-up may be due to lack of eye health education, which prevents them from understanding the reasons for the referral letter or the diagnosis and treatment.

*‘Parents don’t necessarily understand the implication of having a lazy eye for example; there is an element of lack of education on [eye health] for the parents to appreciate potential issues for the eyes’ [P12]*.

*‘If the potential problem has been identified in school today, and the letter comes home, if they haven’t noticed the problem I wonder whether they are not particularly convinced that there is a problem […] I wonder whether because they were not there, this letter might get easily ignored’ [P11]*.

This perception is reinforced when during clinical encounters parents tend to be surprised by the diagnosis, as they had not previously noticed a problem. This is particularly common with parents of children with amblyopia, a condition in which one eye has reduced vision but which does not result in any visible disability in day-to-day life:

*‘[these parents are] surprised to hear that their child has a lazy eye, and wondering why their child needs to wear glasses, because he shows no problem’ [P6]*.

In these clinical encounter professionals need to find a balance between the need to reinforce the medical explanation and the impact which this may have on families. In some of these cases, vision specialists felt that they needed to re-assure parents because

*‘once parents see that [a vision problem in their child], they are often quite shocked and feel guilty about it, like they should have done something about it, so you have to reassure them that there is nothing they could have done’ [P13]*.

Orthoptists may find themselves explaining to parents that

*‘[their] child doesn’t know he can’t see that well. And you don’t know as a parent, until we cover one eye up and demonstrate it. So, it’s fine that you didn’t know’ [P3]*.

Besides, some parents may get upset with the results, as they always thought their child was ‘perfect’ [P7]. Vision specialists believed this reaction could also be related to the parents’ own experience of having to wear glasses.

*‘Parents grew up in an age when wearing glasses was quite a stigma, and if they had negative experience with glasses or squint when they were children, they are more reluctant if their children need treatment’ [P9]*.

There have also been cases where parents deny the existence of any problem in their child. Eye care professionals reported struggling with these parents, especially as to how to ensure treatment will be followed. A child may argue:

*‘why would I need to wear the glasses if I can’t see any better?’ And if the parents don’t understand that, they’ll think ‘yes, why does he have to wear glasses?’ [P6]*.

When facing parents who are denying the child’s problem, some vision specialists claimed that re-testing the child in front of them and showing them where the problem is occurring is helpful.

*‘There is something quite striking about showing parents that they see well with one eye [but] can only see the biggest letter with the other. It speaks for itself’ [P3]*.

Finally, other more socio-cultural factors can play a role in the development of these clinical encounters. Many areas in the UK have ethnically diverse populations, and vision specialists have found that in some cultures and in some people from lower socio-economic backgrounds, glasses or patches are still perceived as a social stigma and parents are more reluctant to have their child wearing them.

*‘I think certain cultures are less keen on spectacles still. Probably the Asian population, even South-east Asian. Even when other people in their family may already be wearing glasses, they don’t want their child to wear glasses, because there is an impression that their child might become dependent on the glasses or their eyes might get worse’ [P10]*.

Or:

*‘African [parents] regard it as a form of physical handicap and are not prepared to recognise that’ [P13]*.

### Professional-parent-child Relation as Key to Improve Child Eye Health

This second theme centres around the relationships between health professionals, young patients and their carers which were central in enhancing treatment uptake and follow-up.

#### Developing trust through good communication

When working in children’s vision care, the type of relationship established during the first assessment can have consequences beyond the visit itself, given the importance of early treatment in childhood eye disorders. A key element which eye care professionals agree is fundamental in the relationship with parents and children is trust:

*‘the parents need to be on board with you. Otherwise [children] won’t wear the glasses or they won’t put the patch on, because the parents have to do it’ [P5]*.

*‘If they feel you are not there to criticise them, I think they do trust you a little bit more. So, it’s about establishing a rapport with them’ [P11]*.

Vision specialists believed that good communication is central in building a trustworthy relationship with the parents and this communication needs to be tailored to the type of patient they are dealing with, both the child and the parents.

*‘Everyone has a different style. Because I’m a mum, then actually it does become a mum-to-mum relationship. If it’s a professional mum, that often doesn’t work’ [P3]*.

Lay terms are preferred;

*‘avoid any kind of scientific terminology that they can’t understand. So, you wouldn’t use amblyopia, you would say lazy eye, or poor vision’ [P14]*.

*‘I try to be fun, and informal, make it like a bit of a game, you know, make it less scary as possible’ [P1]*.

Professionals also adopt lay explanatory frameworks to ‘anchor’ clinical explanations within the social and cultural understanding of carers (Sanders & Roberts 2017). In some cases, professionals explained the treatment to the child hoping the parents would understand it better:

*‘I need you to be a pirate for two hours in the evening, so when you come home from school you need to put the patch on. It’s not just for the child, as I don’t expect them to come home and do it, but it’s a simple way to explain it also to the parents without insulting them’ [P11]*.

Consistency in the messages delivered is also perceived as key in developing a trustworthy relationship with carers. This, in turn, can enhance acceptance of the vision problem and the treatments proposed, and increase the likelihood of parents’ follow-ups.

*‘Often parents ask the same questions. It’s when they start hearing different things that parents are not sure on what to believe and if what we are doing is correct’ [P10]*.

Especially in hospital settings, where

*‘parents won’t see the same person at every visit [it is fundamental to be] consistent in what we say’ [P8]*.

As for dealing with families whose English is not their mother tongue, language barriers were not perceived as problematic, given the availability of interpreters and the fact that most non-English speaking parents may

*‘come with a family member or friend to help them’ translate [P1]*.

#### Involving children and parents in eye care

Some vision specialists believed it is important to involve the child as much as possible, both during the visit and for the choice of treatment such as glasses.

*‘Children are naturally curious […] You can bring them along, and keep them sufficiently interested, […] try not to frighten them, use equipment that is suitable for them, that is going to keep them interested and then, keep talking to them all the time’ [P6]*.

As for the treatment,

*‘if you get the child involved in choosing the glasses, they are more likely to wear them’ [P5]*.

Nonetheless, concordance with the treatment can be quite challenging for some families. Most difficulties seem to arise when the glasses do not appear to improve the child’s vision.

*‘Like in squint, the child may have no symptoms of that without glasses. Put the glasses on, and now the eyes are straight, but again no symptoms. Their view would be same’ [P6]*.

Another potential barrier was identified in parents expressing concern that their child may be bullied at school.

*‘Wearing glasses is something that everybody else sees, so it is a problem for potential stigma or bullying’ [P9]*.

However, nowadays children react more positively when it comes to wearing glasses, and professionals reported they may be disappointed when told they should not wear them:

*‘more kids these days probably are upset if you tell them they don’t need glasses’ [P1]*.

This is felt to be related to the fact that fashion and aesthetics around them have changed.

*‘Some children are unhappy about not having glasses prescribed because I think they are becoming more fashionable’ [P5]*.

*‘Glasses are now much nicer than they might have been 15–20 years ago. […] Now they have characters’ frames, like Disney frames’ [P14]*.

In many cases, their peers wearing glasses is a favourable factor too

*‘sometimes children come for eye examination hoping to get glasses, because their friend has a nice pair of glasses and they think they look nice’ [P6]*.

#### Person-centred clinical advice

When the assessment confirms a vision problem for which treatment is advised, the challenge becomes how to support young children who tend to struggle with adherence:

*‘when you are five years old, it’s hard to explain to a child why they should be wearing glasses’ [P7]*.

To support parents with following the treatment recommendation, eye care professionals suggest the treatment should be tailored to the family’s need.

*‘If we say to patch three to four hours, and they ask at which time, we’ll ask them “what time do you think it would be best for your child?”’ [P10]*.

Providing meaningful advice has also been useful to support parents who are struggling. For example, with patching:

*‘tell the child “it will come off at tea time,” so if that is at four o’clock, on the first day put it on at a quarter to four, as the child has no idea or concept of time, and then it would come off at four. And then on day two, tell him it’s coming off at tea time, but put it on at half past three’ [P4]*.

Another example is to associate glasses with fun moments, so that the child

*‘only could play when he has the glasses on. And they’ll learn quickly’ [P7]*.

Finally, to ensure concordance with the treatment during school hours, school teachers are seen as helpful in providing additional support. Thus, professionals encourage parents to inform the teacher, when possible, about the vision treatment which the child should follow:

*‘If the child isn’t wearing glasses, then the teacher is going to be the first person to know. I mean, the child could walk into school with the glasses on, take them off, put them in the school bag, and then leave them off all day, and put them back again on their way out. And the parents may think they have been wearing their glasses all day’ [P6]*.

The findings emerging from this study have provided insights into how children’s eye care is organised and where the major challenges lie. The vision specialists in this study shared similar views and concerns over the distinct factors which can play a role when examining children compared to adults’ eye care, and the factors that can affect referral uptake, service use and concordance with treatment. Parents’ concordance with the treatments is mediated by their knowledge and understanding of vision problems and their view as lay expert on their child, their needs and the practicality of the recommended treatment. Thus, professionals need to draw on different strategies to respond to the attitudes of the families they engage with. A successful balance of these different perspectives during the clinical encounter can help provide a more child- and family-centred care and improve children’s vision and treatment uptake.

## Discussion

This study presented the views of vision specialists towards the challenges of treating children with vision problems and the strategies adopted to overcome them. Based on these findings, three strategies are suggested as a way to improve children’s eye health and care.

First, awareness of eye health should be raised in parents and carers and its importance for child well-being and development. This can be promoted through awareness raising campaigns, shown to be effective in other health behaviours (Wakefield et al. 2014). Moreover, culturally sensitive health education approaches, which take into account parents’ beliefs and perceptions of eye health, can enhance awareness around certain health topics (Crawford et al. 2015). This finding was highlighted by vision specialists in this study and it is supported in the wider published literature. For instance, previous research has highlighted limited knowledge in the general public of the differences between vision screening and a comprehensive eye test (Su et al. 2013; Lyons et al. 2011; Schmalzried et al. 2015; Frazier et al. 2012). This lack of understanding and its impact on Did-Not-Attend (DNA) rates should not be underestimated. In fact, eye health is most commonly not considered a priority by the general public (Williams et al. 2013; Su et al. 2013; Noma et al. 2011; Patel et al. 2006) and eye health care seeking behaviour in adults is generally symptom-driven (Frazier et al. 2012; Leamon et al. 2014; Ramai et al. 2015; Shickle et al. 2015; Patel et al. 2006; Johnson et al. 2011).

This could potentially explain the high rates of non-attendance in some areas of England (Bruce & Outhwaite 2013) and the parents’ lack of acceptance described by vision specialists in this study, when a child is firstly diagnosed with an eye disorder. This is particularly evident in children diagnosed with amblyopia, as the lack of symptoms makes it difficult for parents to detect any problem and could influence their willingness to follow-up referrals. Improving parents’ understanding and knowledge of children eye health and its care should thus be actively promoted.

Lay health beliefs should also be taken into consideration if effective vision care education is to be delivered to parents and carers. Professionals claimed that ‘stigma’ was a potential barrier to concordance with treatment. The parents’ need to maintain ‘normality’ and refusal to see their child as having ‘impairments’ may result in lack of acceptance of diagnosis and treatment. This is in line with research from outside the UK reporting that cultural and social factors impact on referral and treatment uptake (Truong & Fuscaldo 2012; Senthilkumar et al. 2013; Aldebasi 2013).

Second, a more personalised referral communication pathway could support families in accessing eye care. For instance, as suggested by a participant in the study, direct telephone contact with parents prior to the first appointment could improve attendance. In some areas orthoptists contact patients who do not attend (DNA) appointments by telephone and although time-consuming, it was found to be successful, reaching up to 90% of attendance to follow-ups, according to one participant in this study. This would require additional administrative support to assist with contacting parents and its cost-effectiveness needs to be evaluated. Nonetheless, direct telephone follow-up could help parents navigate the eye care system, leading to increased willingness to access care and engage with treatments (Pizzi et al. 2015; Neville et al. 2015). If proven beneficial, specific recommendations on direct outreach to patients could be incorporated into guidelines for non-attenders (Arai et al. 2015). Moreover, direct contact by telephone with parents could also prevent inequalities generated using written language for those families with lower literacy skills. Although participants in this research did not perceive language as a barrier, the level of literacy of families where English was not their first language could hinder their capacity to fully understand the referral letter and the underlying clinical explanation. A study (Wahl et al. 2011) showed that parents from ethnic minority backgrounds may be able to communicate verbally but unable to read or write.

Third, better coordination of the pathways between community services and hospital care could contribute to families’ understanding of vision care services and enhance their intention to uptake referral and follow up treatments. Professionals in this study reported that trustworthy relations and consistency in the communication of key messages are central to encouraging both the child and the family to accept the diagnosis and treatment. Teamwork and sharing of patient notes are fundamental to providing a comprehensive service for children and their family. However, some community optometrists expressed concern that they do not receive adequate feedback from the hospital once they have referred a child. It could be argued that the limited direct dialogue between professionals working in the community and in hospitals risks divulging conflicting advice to patients and their family, which in turn could influence their uptake of treatment or further visits. A shared responsibility for children’s eye care including vision specialists and primary care providers needs to be considered (Ewing et al. 2016). Recent research on integrated primary and secondary care in communities has shown that these models can work (Montgomery-Taylor et al. 2016, Woodman et al. 2015). A more integrated care pathway between the hospital and primary care services in the community could therefore support the provision of a greater holistic approach.

## Conclusion

This study explored the views of healthcare professionals towards the current care model of children’s vision treatment in England using a qualitative design. These findings can inform the development of future interventions tailored at raising awareness of the importance of childhood vision disorders and at increasing referral and treatment uptake. As with all qualitative studies these results are not statistically generalisable beyond our study location and population, however, it could be expected that many of the views expressed by the ophthalmic professionals would be similar across different regions of the UK, particularly in areas of deprivation and in multi-ethnic communities. Further research is required to investigate the views of families and other care providers such as GPs to identify ‘system barriers’ to accessing and engaging with vision care in this young population. This will provide further evidence contributing to our understanding of the barriers in children’s eye care and support the development of effective evidence-based packages of child-centred services.
